# Supplementary material for: Clinical isolates of Mycobacterium avium complex reveal an M. intracellulare-associated IL-17/neutrophilic pulmonary immune program in a murine disease model
Source: mSphere. 2026 Sep 21;11(10):e00398-26. doi: 10.1128/msphere.00398-26 (PMC13711828; doi:10.1128/msphere.00398-26)
Supplement: Supplementary file 1 [file msphere.00398-26-s0001.pdf]

A

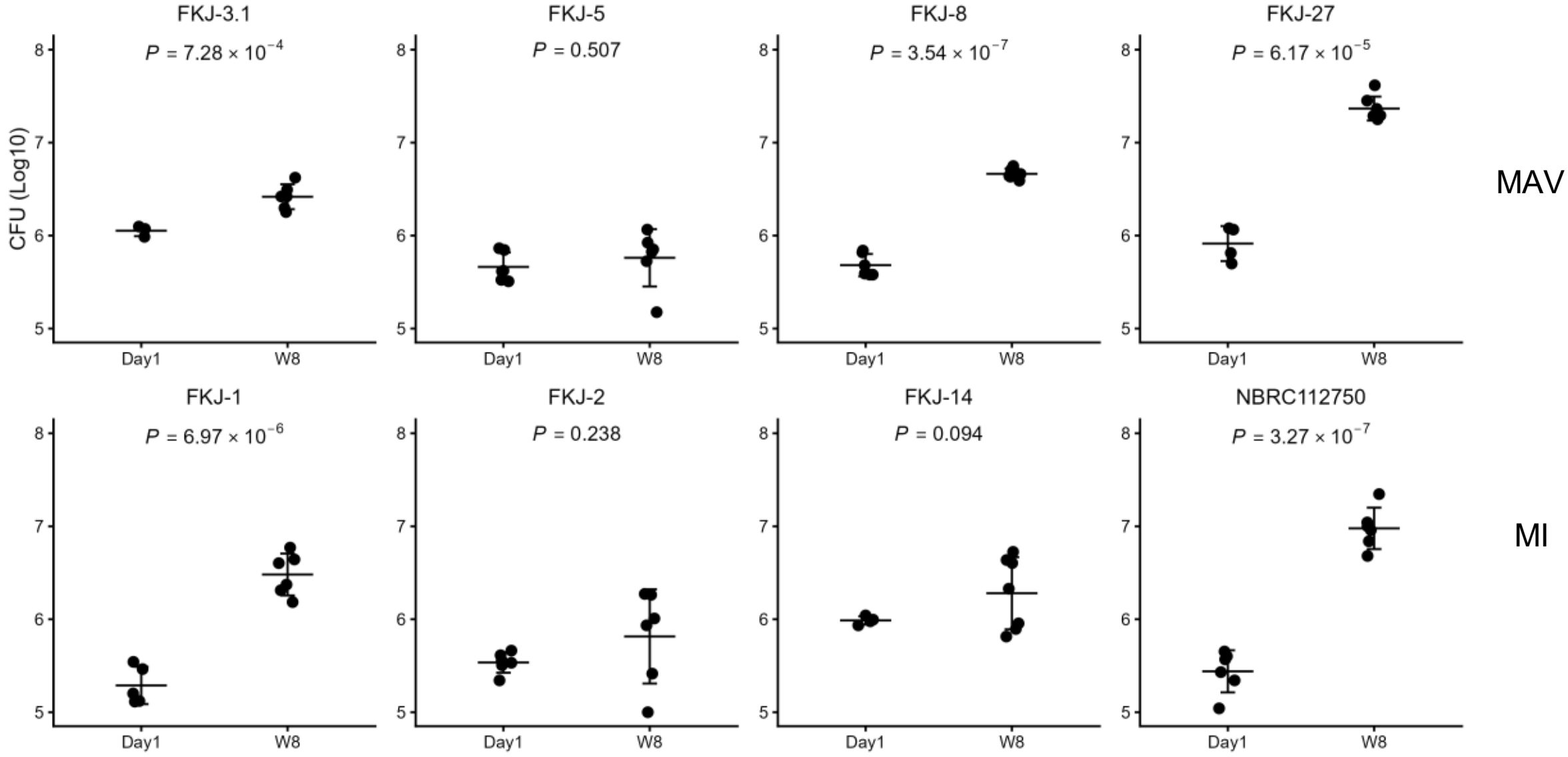

B

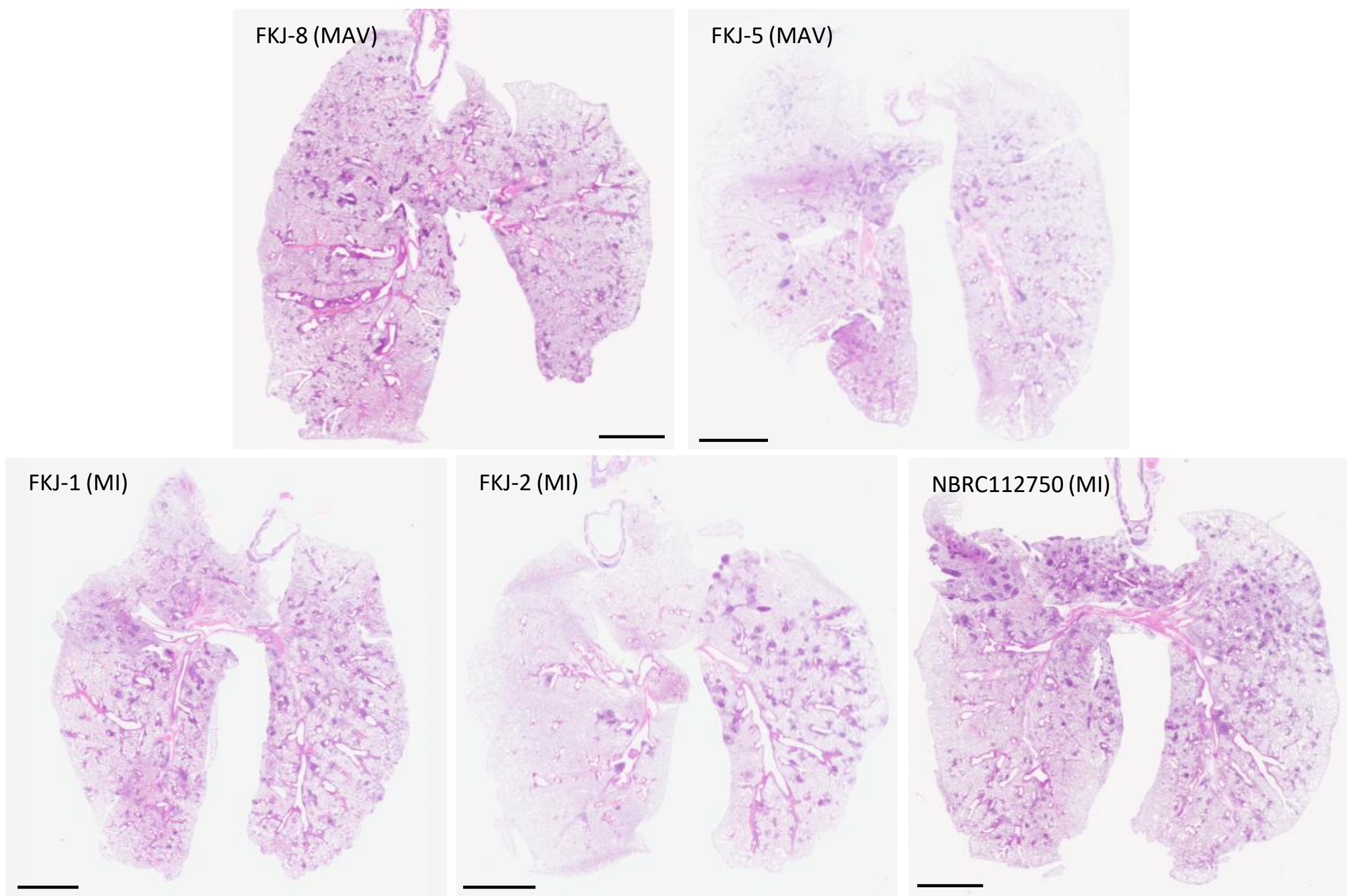

FIG S1 (A) Pulmonary bacterial burdens in BALB/c mice infected with clinical MAC isolates. Lung bacterial burdens were determined by CFU assay on Day1 and 8W p.i. (B) Representative hematoxylin and eosin-stained images of BALB/c mouse lungs infected with the indicated MAC isolates at 8 weeks p.i. Scale bars, 2.5 mm. These data include results for three isolates newly examined in the present study (FKJ-3.1, FKJ-14, and FKJ-27) and the other five isolates obtained in our previously published infection experiments (20).

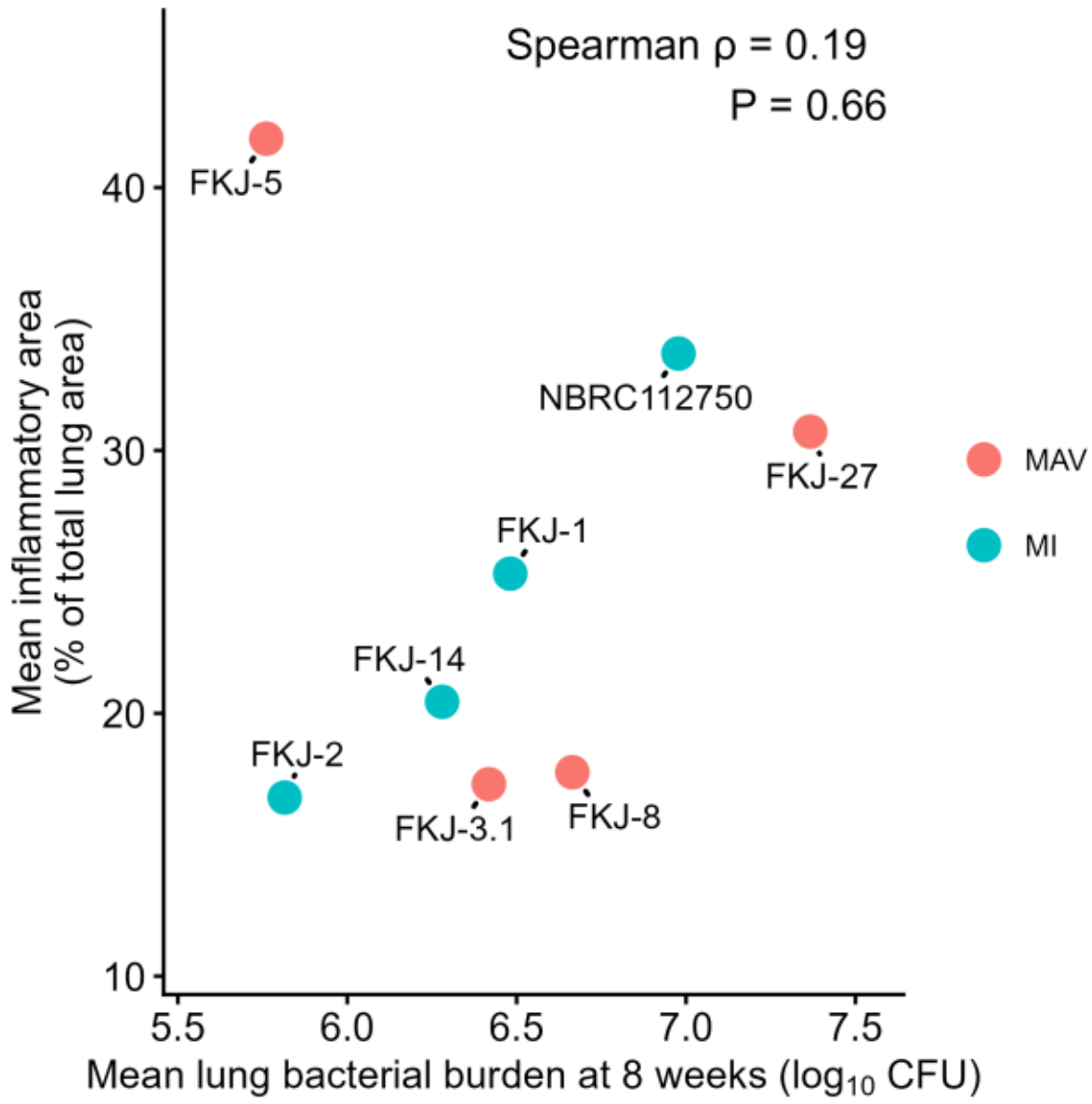

FIG S2 Association between pulmonary bacterial burden and inflammatory area across MAC isolates. The mean pulmonary bacterial burden at 8 weeks p.i. was plotted against the mean proportion of inflammatory area in the total lung area for each isolate. Each symbol represents one MAC isolate and is colored according to species. Mean values were calculated from 6–7 mice per isolate for pulmonary bacterial burden and 2 mice per isolate for histological analysis. The association was assessed using Spearman's rank correlation. No significant correlation was observed between pulmonary bacterial burden and inflammatory area (Spearman's ρ = 0.19, P = 0.66).

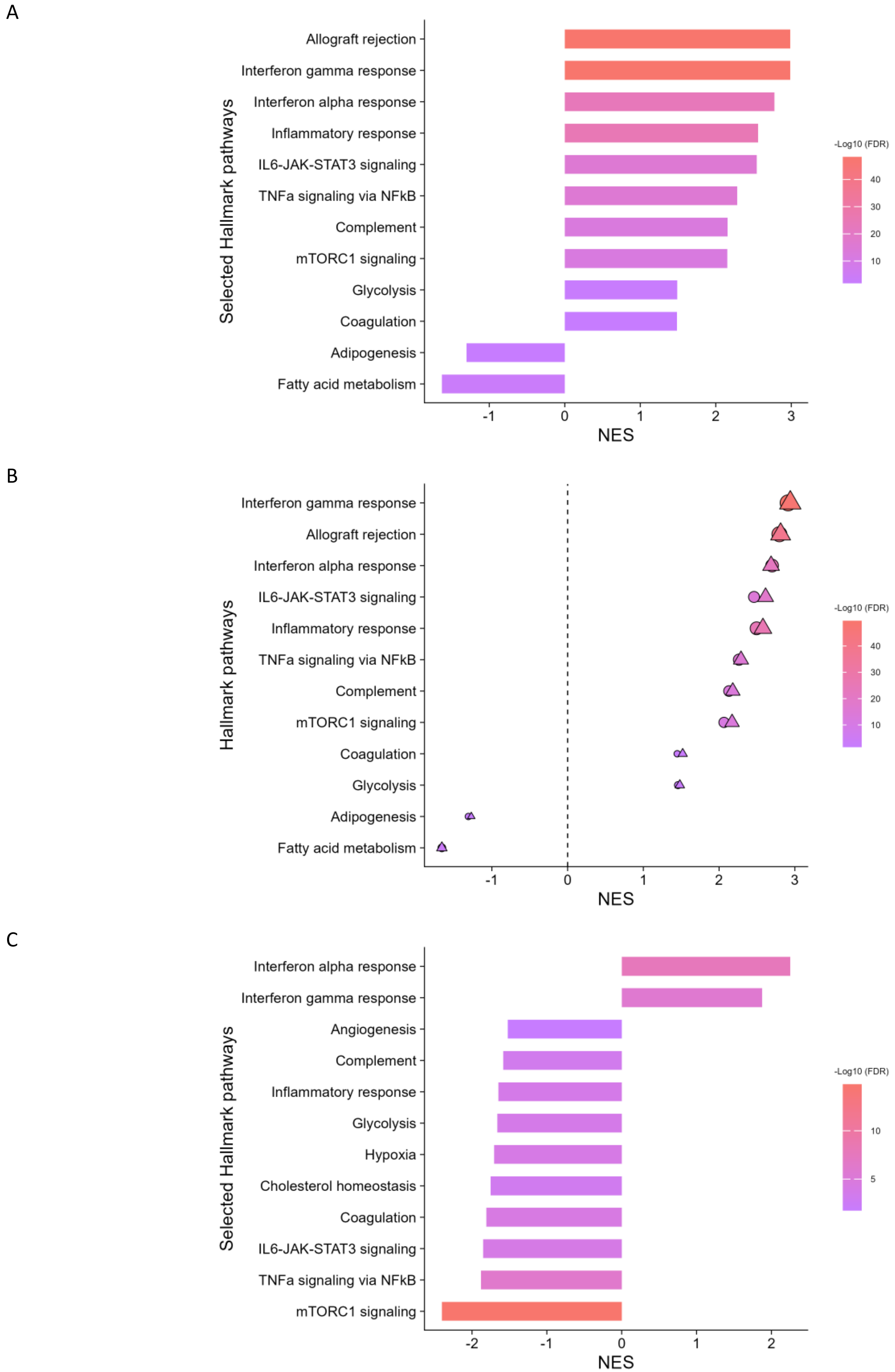
A
Selected Hallmark pathways
Allograft rejection
Interferon gamma response
Interferon alpha response
Inflammatory response
IL6-JAK-STAT3 signaling
TNFa signaling via NFkB
Complement
mTORC1 signaling
Glycolysis
Coagulation
Adipogenesis
Fatty acid metabolism
-1
0
1
2
3
NES
-Log10 (FDR)
40
30
20
10
B
Hallmark pathways
Interferon gamma response
Allograft rejection
Interferon alpha response
IL6-JAK-STAT3 signaling
Inflammatory response
TNFa signaling via NFkB
Complement
mTORC1 signaling
Coagulation
Glycolysis
Adipogenesis
Fatty acid metabolism
-1
0
1
2
3
NES
-Log10 (FDR)
40
30
20
10
C
Selected Hallmark pathways
Interferon alpha response
Interferon gamma response
Angiogenesis
Complement
Inflammatory response
Glycolysis
Hypoxia
Cholesterol homeostasis
Coagulation
IL6-JAK-STAT3 signaling
TNFa signaling via NFkB
mTORC1 signaling
-2
-1
0
1
2
NES
-Log10 (FDR)
10
5

FIG S3-1

FIG S3 GSEA of MAC-infected mouse lungs. (A) GSEA of infected lungs (MAV and MI) compared with uninfected control lungs. Bar length indicates NES, with positive and negative NES values representing enrichment in infected and uninfected lungs, respectively. Color indicates the $-\log_{10}$-transformed FDR.

(B) GSEA of lungs infected with MAV or MI compared with uninfected control lungs. Selected Hallmark gene sets enriched in MAV- or MI-infected lungs compared with uninfected control lungs are shown. Circles and triangles indicate MAV- and MI-infected lungs, respectively. Point size and color indicate the $-\log_{10}$-transformed FDR.

(C) GSEA comparing lungs infected with FKJ-1 and FKJ-8. The same Hallmark gene sets in Fig. 2B are shown for the comparison between FKJ-1- and FKJ-8-infected lungs. Bar length indicates the NES, with positive and negative NES values representing enrichment in FKJ-8- and FKJ-1-infected lungs, respectively. Color indicates the $-\log_{10}$-transformed FDR.

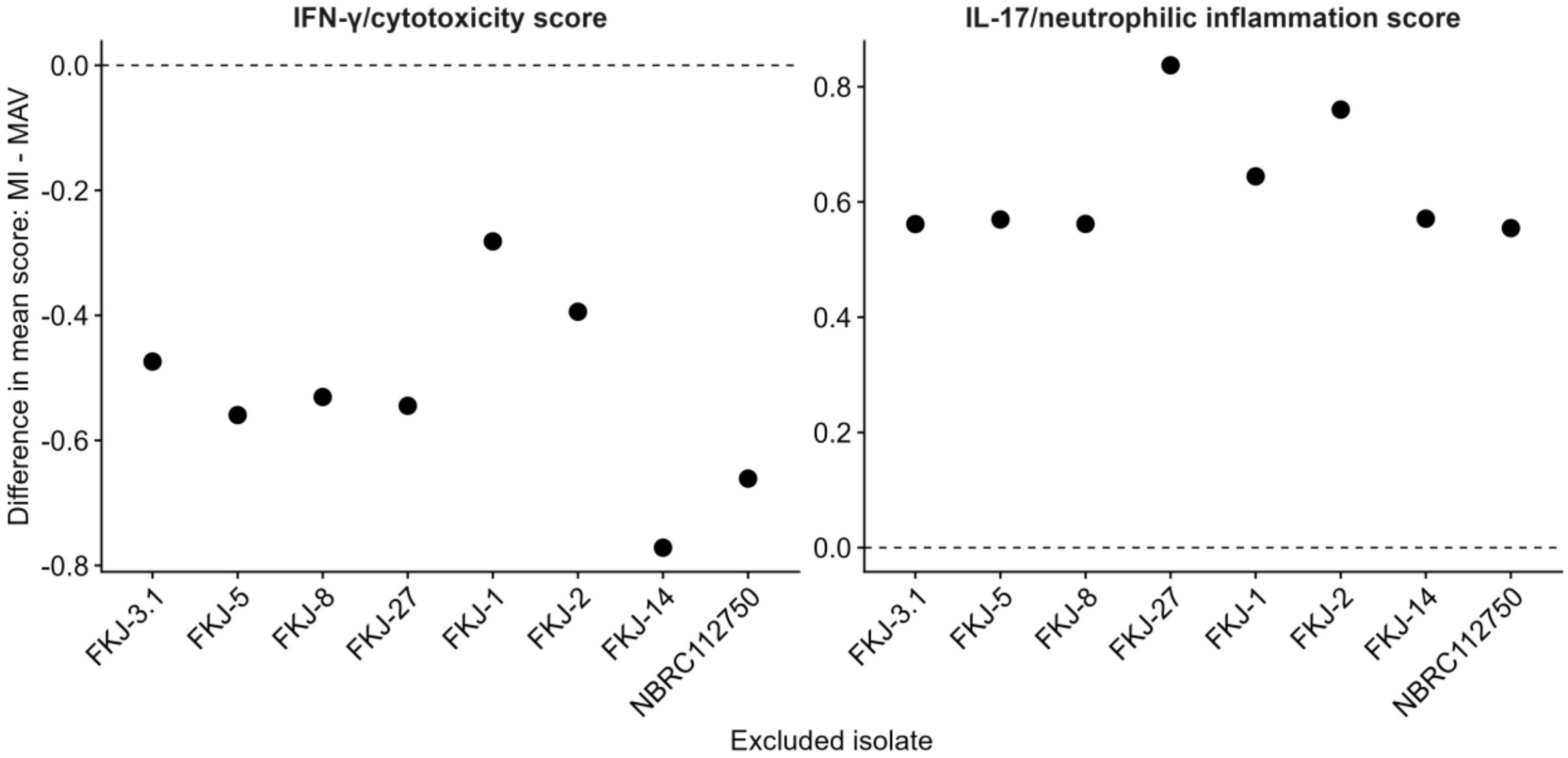

FIG S4 Leave-one-isolate-out analysis of isolate-level mean signature scores. Each point shows the difference in mean score between MAV and MI isolates after excluding one isolate. Positive and negative values indicate higher scores in MI- and MAV-infected lungs, respectively.

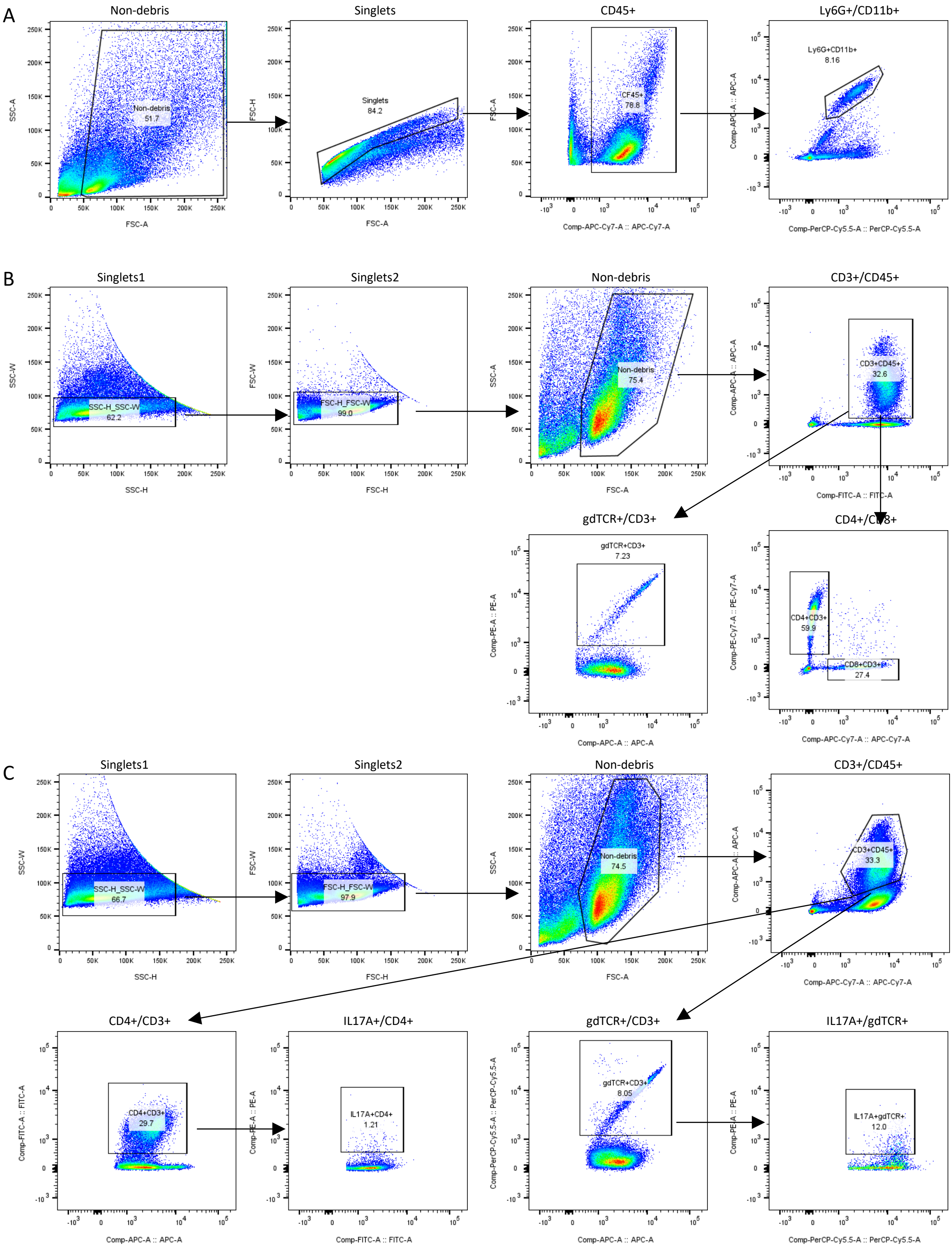

FIG S5. Gating strategy for FCM. (A) Neutrophils, (B) CD4⁺ T, CD8⁺ T, and γδ T cells, (C) IL-17A⁺ cells among CD4⁺ T cells and γδ T cells.

FIG S5

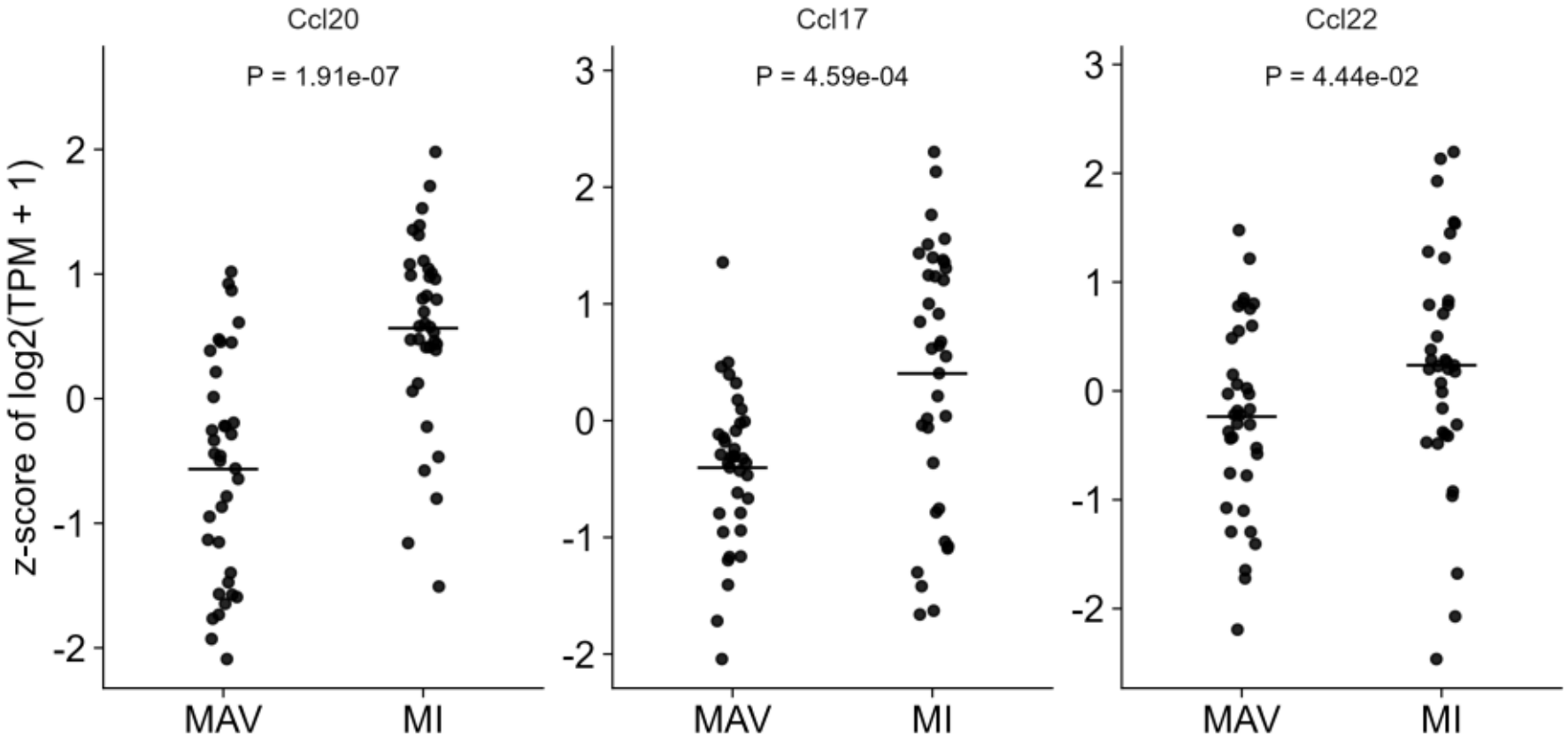

FIG S6 Expression of chemokine genes associated with $CD4^+$ T-cell recruitment in MAC-infected lungs. Expression levels of *Ccl20*, *Ccl17*, and *Ccl22* in MAV- and MI-infected lungs were analyzed using the bulk RNA-seq data. Values are shown as *z*-scores calculated from log2(TPM + 1) values for each gene. Each data point represents one mouse, and horizontal bars indicate the mean. *P* values were determined using Welch's *t*-test.

FIG S6

A

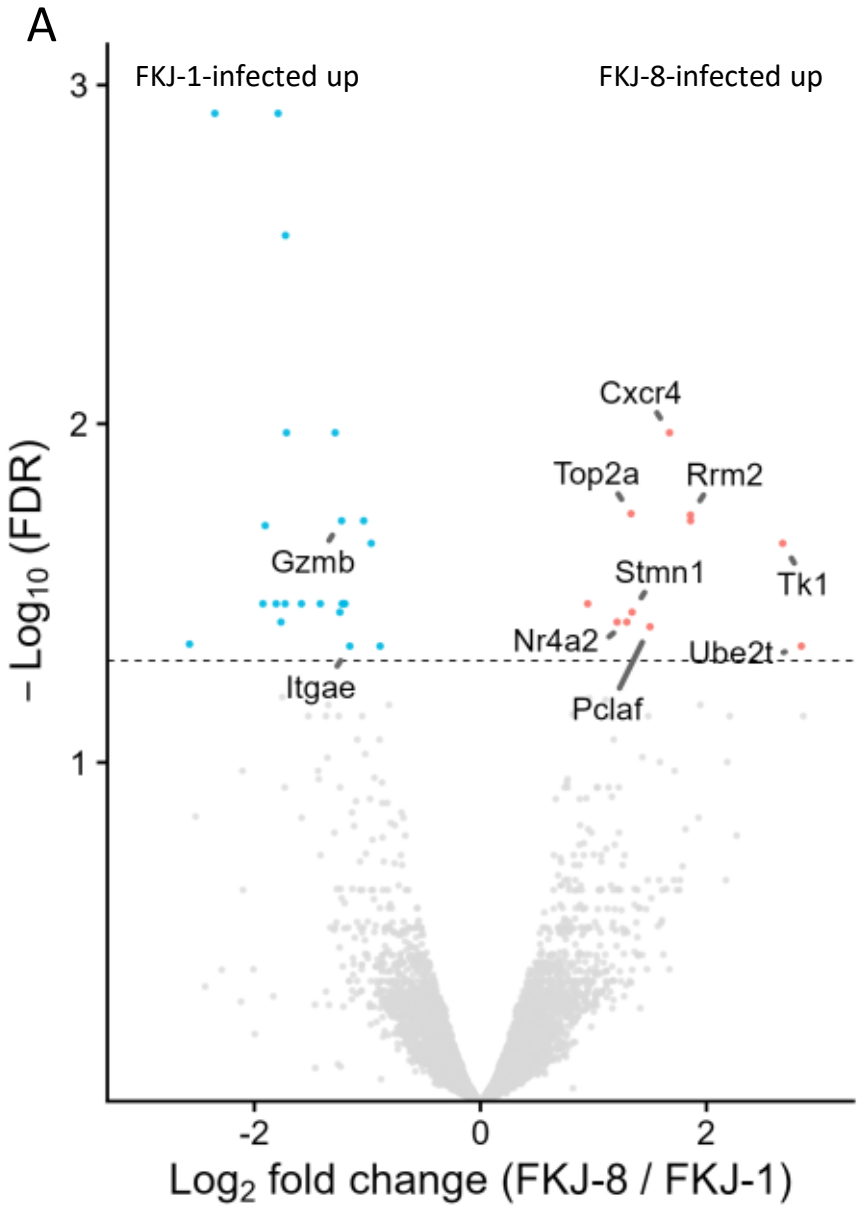

B

Predefined signature	Genes tested	NES	FDR	Interpretation
Cytotoxicity	11	−1.20	0.299	Not significant
CD8 effector	13	−1.11	0.335	Not significant
CD8 memory	12	1.38	0.23	Not significant
Proliferation	20	2.65	7.09×10^{-11}	Higher in FKJ-8
T-cell activation	19	1.92	0.0022	Higher in FKJ-8

FIG S7 Functional-state analysis of pulmonary CD8 T cells in FKJ-1- and FKJ-8-infected lungs.

(A) Volcano plot of sample-level pseudo-bulk differential expression analysis of bona fide $CD3^+CD8^+$ αβ T cells identified from the scRNA-seq dataset. Three biological replicates were analyzed for each infection group using edgeR. Positive $\log_2$ fold-change values indicate higher expression in FKJ-8-infected lungs, whereas negative values indicate higher expression in FKJ-1-infected lungs. Red and blue dots indicate genes significantly upregulated in FKJ-8- and FKJ-1-infected lungs, respectively. Genes with an FDR of less than 0.05 were considered differentially expressed. Representative significant genes associated with CD8 T-cell proliferation, activation, migration, or effector function are labeled.

(B) Gene set enrichment analysis of five predefined CD8 T-cell functional signatures using genes ranked by the signed edgeR quasi-likelihood test statistic. Positive normalized enrichment scores indicate enrichment in FKJ-8-infected lungs, whereas negative scores indicate enrichment in FKJ-1-infected lungs. An FDR of less than 0.05 was considered statistically significant. The signatures and their selection rationale are provided in Table S10.
